# Effects of Topical Steroids and Non‐Steroidal Anti‐Inflammatory Drugs on Gastrointestinal Bleeding in Rats

**DOI:** 10.1002/vms3.70962

**Published:** 2026-04-24

**Authors:** Harun Cinar, Latif Emrah Yanmaz, Ozlem Ozmen, Aybike Ozbeyli, Beyza Bektas

**Affiliations:** ^1^ Department of Surgery, Faculty of Veterinary Medicine Burdur Mehmet Akif Ersoy University Burdur Turkey; ^2^ Department of Pathology, Faculty of Veterinary Medicine Burdur Mehmet Akif Ersoy University Burdur Turkey

**Keywords:** absorption, diclofenac, gastrointestinal, ocular, prednisolone, rats

## Abstract

**Objective:**

This study aimed to investigate the potential systemic gastrointestinal (GI) effects of short‐term topical administration of ocular steroids and non‐steroidal anti‐inflammatory drugs (NSAIDs) in rats.

**Animals:**

A total of 56 adult male Wistar rats were assigned to seven treatment groups (*n* = 8 for each group), receiving topical applications of saline, prednisolone acetate, dexamethasone phosphate, loteprednol etabonate, ketorolac tromethamine, nepafenac or diclofenac sodium for 1 week.

**Methods:**

GI bleeding was assessed using faecal occult blood tests, gastric and duodenal tissues were analysed histopathologically, and endoscopic evaluations were performed.

**Results:**

Positive faecal occult blood results were observed in the prednisolone acetate group (3/8; 37.5%), dexamethasone phosphate (2/8; 25%), diclofenac sodium (1/8; 12.5%) and loteprednol etabonate (1/8; 12.5%). No positive results were recorded in the ketorolac tromethamine, nepafenac, or saline groups (0%). Differences between groups were not statistically significant (*p* > 0.05). No significant within‐group differences between baseline and post‐treatment results were detected (*p* > 0.05). Endoscopic scores ranged from 0 to 1 across all groups, and histopathology revealed no abnormalities.

**Conclusions and clinical relevance:**

Short‐term topical ocular steroid and NSAID treatments were not associated with statistically significant systemic gastrointestinal alterations under the conditions of this study.

## Graphical abstract

Short‐term topical ocular steroids and NSAIDs did not result in statistically significant gastrointestinal bleeding or histopathological injury in healthy rats. Limited faecal occult blood positivity was observed in some steroid‐treated animals but was not accompanied by macroscopic or microscopic evidence of mucosal damage

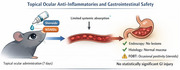

## 1 Introduction

Topical ocular medications are fundamental to ophthalmic therapy, facilitating precise delivery of pharmacological agents to the eye (Souza et al. 2014). Steroids and non‐steroidal anti‐inflammatory drugs (NSAIDs) are commonly prescribed for various ocular conditions including inflammatory disorders, allergic conjunctivitis and postoperative pain management (Salinger et al. 2019; Gaynes and Fiscella 2002). Although the primary aim of local administration is to confer therapeutic benefits while minimizing systemic exposure, apprehensions persist regarding their potential impact on the gastrointestinal (GI) tract (Pytrus et al. 2022).

Systemic absorption of topically administered ocular medications can occur through various routes, including conjunctival and nasal mucosa, leading to distribution into the systemic circulation and potential systemic adverse effects (Yang and Lockwood 2022). Systemic effects of oral steroids and NSAIDs are well‐documented (Domper Arnal et al. 2022; Cooper et al. 2019; Eren et al. 2016), yet limited research has explored the impact of topical ocular medications on GI health. A recent study in dogs has warned of an increased risk of GI bleeding associated with the use of ophthalmic NSAIDs (Van Vertloo et al. 2023). Therefore, this experimental study aimed to investigate the effects of topically administered steroids and NSAIDs on GI bleeding in rats, providing insights into their potential systemic effects. It was hypothesized that short‐term topical ocular administration of steroids or NSAIDs would not induce significant gastrointestinal bleeding or histopathological alterations.

## 2 Materials and Methods

### 2.1 Ethical Statement

All procedures were employed in accordance with the Guide for the Care and Use of Laboratory Animals (National Resources Council 2011) and were approved by the Burdur Mehmet Akif Ersoy University Local Board of Ethics Committee for Animal Experiments (Decision no: 116/1205).

### 2.2 Animals

The study was conducted on 56 adults male Wistar rats (body weight 250–300 g) obtained from the outbred stock maintained at the Burdur Mehmet Akif Ersoy University Laboratory Animals Research Centre, and all experiments were conducted within the same facility. The animals were kept under standard conditions (12/12‐h light/dark cycle, free access to food and water), in accordance with the requirements of the Directive 2010/63/EU of the European Parliament and of the Council on the protection of animals used for scientific purposes.

### 2.3 Study Design

The study included eight rats per group, each subjected to topical eye drop applications of different solutions. The treatments were as follows: saline (Poliflex 500 mL, Polifarma, Tekirdag, Turkey), prednisolone acetate (Pred‐forte 1%, 50 mg/5 mL, Allergan, Mayo, Ireland), dexamethasone phosphate (Onadron Simple 0.1%, 5 mg/5 mL, IE Ulagay, Istanbul, Turkey), loteprednol etabonate (Lotemax 5 mg/mL, Bausch & Lomb Inc., Florida, USA), ketorolac tromethamine (Acular LS 0.4%, 20 mg/5 mL, Allergan, Texas, USA), nepafenac (Apfecto 0.1%, 5 mL/5 mg, World Medicine, Istanbul, Turkey), and diclofenac sodium (Inflased 0.1%, 5 mg/5 mL, Mefar, Istanbul, Turkey). Eye drops (10 µL) were administered to both eyes six times in a day over 1 week. The regimen of the treatments was in line with previous findings demonstrating how to apply non‐steroid and steroid treatments in humans (McGhee et al. 2002).

### 2.4 Endoscopic Evaluation

Gastrointestinal endoscopy was performed on the final day of the study under general anesthesia with intraperitoneal administration of 50 mg/kg ketamine and 5 mg/kg xylazine using a veterinary endoscope (Hopkins 30°, Karl Storz, Germany). A flexible endoscope (diameter: 2.7 mm) was inserted into the stomach and duodenum to evaluate mucosal integrity. Endoscopic findings were assessed using a modified gastrointestinal injury scale (Koelink et al. 2018), ranging from 0 (normal) to 4 (severe). Scoring was conducted in a blinded manner by a single evaluator who was unaware of the treatment groups.

### 2.5 Immunochromatographic Faecal Occult Blood Test

Fresh faecal samples were obtained from each rat at baseline and at final day of the study. Commercially available immunochromatographic faecal occult blood tests (Laboquick, Koroglu Medical Devices Ltd, Izmir, Turkey) were employed for analysis. The sample collection device's stick was inserted into the faeces at four different sites and promptly placed into the extraction buffer solution. The mixture was thoroughly shaken to ensure proper extraction. Subsequently, three drops of the extracted sample were loaded into the sample opening of the test device. Interpretation of results was conducted within 10 min. A single line in the result window of the test device indicated a negative result, while the presence of a double line was considered positive.

### 2.6 Histopathological Analysis

Stomach and duodenum samples were harvested and fixed in 10% buffered formalin. The taken tissue samples were routinely processed by a fully automatic tissue processing equipment (Leica ASP300S, Leica Microsystem, Nussloch, Germany) and embedded in paraffin wax. Five‐micron thickness sections were taken from the paraffin block levels by a fully automatic rotary microtome (Leica 2155, Leica Microsystem, Nussloch, Germany). After drying, the preparations were passed through alcohol and xylol series and stained with Harris hematoxylin‐eosin (HE) (Tek‐Path, Izmir, Turkey) mounted with a coverslip, and examined under a light microscope (Zeiss Axioscope 5 trinocular microscope, Carl Zeiss Microscopy GmbH, Jena, Germany). The histopathological examinations were conducted in a blinded manner by a pathologist unaware of the experimental groups. Hyperaemia was defined as mild vascular congestion within the lamina propria without epithelial disruption, haemorrhage, inflammatory infiltration or oedema.

### 2.7 Statistical Analysis

Because no prior rodent data were available, clinically relevant event rates were estimated based on a recent retrospective study in dogs receiving ophthalmic NSAIDs, in which gastrointestinal bleeding occurred in approximately 10.1% of cases (Van Vertloo et al. 2023). Using this incidence as an external reference, a 10% absolute increase was considered the smallest predefined difference of interest. Conventional two‐proportion power calculations (α = 0.05, power = 0.80) indicated that detecting differences of 10.0% versus 0.0%, 12.5% versus 0.0% and 25.0% versus 0.0% would require approximately 73, 58 and 26 rats per group, respectively, whereas detecting the largest difference observed in the present study (37.5% vs. 0.0%) would require approximately 8 rats per group.

Baseline and post‐treatment faecal occult blood test results within each group were compared using McNemar's test. Comparisons of the proportions of positive results between treatment groups were performed using Fisher's exact test. Statistical analyses were carried out using SPSS version 27.0 (IBM Corp., SPSS Inc., IL, USA). In addition, absolute risk differences and their 95% confidence intervals were calculated for each treatment group relative to saline. A *p* value < 0.05 was accepted as the threshold for statistical significance. Because the saline group had zero events, risk ratios were estimated using a continuity correction (Haldane–Anscombe method). Confidence intervals were calculated using the logarithmic (Katz) method. In groups with zero events in both arms, risk ratios were not estimable.

## 3 Results

All 56 rats completed the study without ocular hypersensitivity, conjunctival irritation, or treatment‐related behavioural abnormalities. Baseline faecal occult blood testing yielded negative results in all animals (56/56; 100%). Post‐treatment testing identified positivity in 3/8 rats treated with prednisolone acetate (37.5%), 2/8 treated with dexamethasone phosphate (25.0%), and 1/8 treated with diclofenac sodium (12.5%) and loteprednol etabonate (12.5%). No positive results were recorded in the ketorolac tromethamine, nepafenac, or saline groups (0/8; 0%). In total, 6/56 rats (10.7%) exhibited faecal occult blood positivity after treatment.

The absolute risk difference relative to saline was 37.5% for prednisolone acetate (95% CI: 13.8%–61.2%), 25.0% for dexamethasone phosphate (95% CI: 3.8%–46.2%), and 12.5% for diclofenac sodium and loteprednol etabonate (95% CI: −3.7% to 28.7%). The corresponding risk ratios were 7.0 for prednisolone acetate (95% CI: 1.9–25.9), 4.7 for dexamethasone phosphate (95% CI: 1.2–18.2), and 2.3 for diclofenac sodium and loteprednol etabonate (95% CI: 0.6–8.4). No measurable risk ratio was calculable for ketorolac tromethamine or nepafenac due to the absence of events (Table 1). McNemar's test did not detect significant within‐group differences between baseline and post‐treatment results in any group (all *p* > 0.05).

**TABLE 1:** Post‐treatment faecal occult blood test (FOBT) positivity and risk estimates by treatment group after 1‐week topical ocular administration in rats (*n* = 8 per group).

Group	*n*	Positive, *n* (%)	Absolute risk difference vs. saline % (95% CI)	Risk ratio vs. saline (95% CI)
Saline	8	0 (0.0)	Reference	Reference
Prednisolone acetate	8	3 (37.5)	37.5 (13.8–61.2)	7.0 (1.9–25.9)
Dexamethasone phosphate	8	2 (25.0)	25.0 (3.8–46.2)	4.7 (1.2–18.2)
Diclofenac sodium	8	1 (12.5)	12.5 (−3.7–28.7)	2.3 (0.6–8.4)
Loteprednol etabonate	8	1 (12.5)	12.5 (−3.7 to 28.7)	2.3 (0.6–8.4)
Ketorolac tromethamine	8	0 (0.0)	0.0 (−12.1 to 12.1)*	Not estimable
Nepafenac	8	0 (0.0)	0.0 (−12.1 to 12.1)*	Not estimable

*Note*: Absolute risk differences are reported relative to saline with 95% confidence intervals; risk ratios were estimated using a continuity correction due to zero events in the saline group. *: *p* value < 0.05.

Endoscopic injury scores ranged from 0 to 1. A total of 48/56 rats (85.7%) scored 0, while 8/56 rats (14.3%) scored 1. Mild gastric hyperaemia was recorded in 3/8 rats treated with prednisolone acetate (37.5%), 2/8 treated with dexamethasone phosphate (25.0%), 2/8 treated with diclofenac sodium (25.0%), and 1/8 treated with loteprednol etabonate (12.5%). Hyperaemia was observed in 1/16 rats in the saline group (6.3%) and was absent in the ketorolac tromethamine and nepafenac groups. No ulcers, erosions, petechiae, haemorrhagic streaks or duodenal lesions were identified.

**FIGURE 1:**
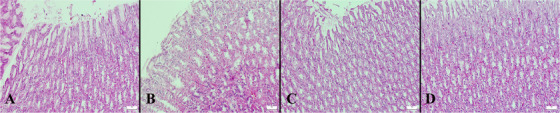
Representative histological appearance of gastric mucosa following 1‐week topical administration of ophthalmic steroids and saline. (A) Prednisolone acetate, (B) dexamethasone phosphate, (C) loteprednol etabonate, and (D) saline. Sections show preserved mucosal architecture without epithelial loss, erosion, haemorrhage, oedema, or inflammatory infiltrates; mild vascular congestion (hyperaemia) may be present in steroid‐treated samples. Haematoxylin and eosin (HE) stain; scale bars = 50 µm.

**FIGURE 2:**
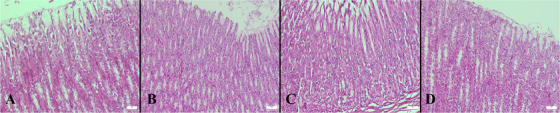
Representative histological appearance of gastric mucosa following 1‐week topical administration of ophthalmic NSAIDs and saline. (A) Ketorolac tromethamine, (B) nepafenac, (C) diclofenac sodium, and (D) saline. Sections show preserved mucosal architecture without epithelial loss, erosion, haemorrhage, oedema, or inflammatory infiltrates. Haematoxylin and eosin (HE) stain; scale bars = 50 µm.

Necropsy revealed no gross abnormalities in any animal. A total of 112 tissue samples (56 gastric, 56 duodenal) were examined histologically. All groups exhibited preserved mucosal architecture without epithelial loss, erosive changes, oedema, haemorrhage, or inflammatory infiltrates. Mild hyperaemia was detected in gastric or duodenal sections of 7/16 rats treated with prednisolone acetate (43.8%), 5/16 treated with dexamethasone phosphate (31.3%) (Figure 1), 3/16 treated with diclofenac sodium (18.8%), and 2/16 treated with loteprednol etabonate (12.5%) (Figure 2). Hyperaemia was absent in all rats treated with ketorolac tromethamine, nepafenac and saline (0/16). All duodenal samples across all groups displayed normal mucosal morphology without structural abnormalities.

## 4 Discussion

This study examined the potential systemic gastrointestinal effects of short‐term topical administration of ophthalmic steroids and NSAIDs in a controlled rat model, addressing concerns previously documented regarding systemic absorption of ophthalmic medications through conjunctival and nasal mucosal routes (Yang and Lockwood 2022). Although topically administered ophthalmic medications may enter the systemic circulation via conjunctival vessels, nasolacrimal drainage and nasal mucosal absorption pathways, thereby partially bypassing first‐pass hepatic metabolism (Farkouh et al. 2016; Vaajanen and Vapaatalo 2017), systemic exposure following therapeutic ocular dosing is generally lower than that observed after oral administration (Patel et al. 2010). In the present experimental model, faecal occult blood positivity was observed only in a limited number of animals receiving prednisolone acetate or dexamethasone phosphate and was not accompanied by endoscopic lesions or histopathological evidence of mucosal injury. Furthermore, no statistically demonstrable differences were detected between treatment groups or between baseline and post‐treatment assessments. These findings suggest that short‐term topical administration under controlled conditions may result in systemic exposure levels insufficient to induce detectable gastrointestinal damage in healthy animals. These observations differ from the retrospective analysis conducted in dogs, which reported a gastrointestinal bleeding incidence of approximately ten percent in animals treated with ophthalmic NSAIDs (Van Vertloo et al. 2023). The discrepancy may be attributable to methodological and population differences, as well as exposure to systemic NSAIDs or glucocorticoids, all of which are established risk factors for gastrointestinal injury (Van Vertloo et al. 2023; Domper Arnal et al. 2022). In contrast, the present study was conducted in rats under standardized experimental conditions, thereby reducing potential confounding influences.

The absence of faecal occult blood positivity in rats treated with ketorolac tromethamine or nepafenac is consistent with previous work describing the favourable systemic safety profile of topical NSAIDs when administered within therapeutic concentrations (Cooper et al. 2019). Systemic NSAID‐induced gastrointestinal injury is primarily mediated through cyclooxygenase inhibition, prostaglandin depletion and impairment of mucosal defence mechanisms (Bjarnason et al. 2018). These mechanisms are unlikely to be triggered to a comparable extent following the limited systemic absorption associated with topical ocular administration.

Histopathological evaluation of gastric and duodenal tissues further supported these findings, revealing no evidence of mucosal haemorrhage, epithelial loss, inflammatory infiltration or oedema in any treatment group. The mild hyperaemia observed in some steroid‐ and NSAID‐treated animals likely reflects transient vascular responses rather than true pathological injury, aligning with earlier studies that reported minimal systemic tissue alterations following topical ocular anti‐inflammatory therapy in experimental and clinical settings (Souza et al. 2014; Pytrus et al. 2022).

The isolated detection of faecal occult blood in the absence of endoscopic or histopathological lesions warrants cautious interpretation. Immunochromatographic faecal occult blood tests are highly sensitive for detecting occult gastrointestinal bleeding; however, their interpretation may be influenced by biological variability and analytical factors (Hundt et al. 2009). False‐positive or transiently positive results have been reported in clinical settings, particularly when bleeding is minimal or intermittent (Symonds et al. 2015). Therefore, isolated faecal occult blood positivity without corroborating macroscopic or microscopic lesions should not be equated with clinically significant mucosal injury.

The absence of gastrointestinal pathology in this study contrasts sharply with the well‐established gastrointestinal complications documented following systemic corticosteroid and NSAID use, including mucosal ulceration, haemorrhage and perforation, as extensively described in the human and veterinary literature (Domper Arnal et al. 2022; Cooper et al. 2019). Although systemic corticosteroid therapy is associated with gastrointestinal adverse effects, systemic exposure following ophthalmic steroid administration is typically minimal and rarely linked to clinically significant systemic complications (Farkouh et al. 2016). The present findings therefore support the advantage of localized ocular delivery, in which drug concentrations remain largely confined to the ocular surface with limited systemic availability, a concept previously demonstrated in pharmacokinetic investigations of ophthalmic agents (Yang and Lockwood 2022).

Despite these strengths, the controlled design used in this study does not entirely exclude systemic absorption, since limited systemic absorption remains possible despite topical administration, as previously detailed in ocular pharmacology studies (Yang and Lockwood 2022). The sample size of the present study was adequate for detecting moderate or pronounced differences in gastrointestinal bleeding but may not identify small increases in risk, similar to those reported in canine retrospective data (Van Vertloo et al. 2023). Furthermore, the short treatment duration limits conclusions to acute exposure, preventing evaluation of cumulative injury that may emerge with prolonged therapy, as suggested in long‐term NSAID toxicity reports (Cooper et al. 2019). The exclusive use of healthy rats without hepatic, renal or gastrointestinal comorbidity also restricts extrapolation to clinical patients who often present with multiple concurrent risk factors known to influence susceptibility to gastrointestinal injury (Domper Arnal et al. 2022). The study was not powered to detect small absolute increases in bleeding incidence below 20%, and rare adverse effects similar to those reported in retrospective canine studies cannot be excluded.

In conclusion, short‐term topical administration of ophthalmic steroids and NSAIDs did not result in statistically significant differences in gastrointestinal bleeding or histopathological alterations compared with saline in healthy rats. Although faecal occult blood positivity was observed in some steroid‐treated groups, these findings were not accompanied by endoscopic or microscopic lesions and did not reach statistical significance. Given the limited sample size and the wide confidence intervals surrounding risk estimates, small increases in gastrointestinal risk cannot be definitively excluded. Further studies incorporating larger cohorts, extended treatment durations and pharmacokinetic measurements are warranted to more comprehensively define systemic gastrointestinal safety profiles of ophthalmic anti‐inflammatory agents.

## Author Contributions

**Harun Cinar**: investigation, methodology, data curation, formal analysis, visualization, software, writing – original draft preparation. **Latif Emrah Yanmaz**: data curation, investigation, methodology, writing – review, editing. **Ozlem Ozmen**: investigation, methodology, writing – original draft preparation. **Aybike Ozbeyli**: data curation, investigation. **Beyza Bektas**: data curation.

## Funding

The authors have nothing to report.

## Ethics Statement

The study received approval from the Burdur Mehmet Akif Ersoy University Local Ethics Committee for Animal Experiments (Approval number: Decision no: 116/1205).

## Conflicts of Interest

The authors declare no conflicts of interest.

## Data Availability

Data available on request from the corresponding author.
